# The sodium/iodide symporter is a nitrate transporter in the human salivary gland

**DOI:** 10.1016/j.redox.2025.103955

**Published:** 2025-11-29

**Authors:** Gaia Picozzi, Leo J.S. Westerberg, Juliane Jurga, Hugo Zeberg, Carina Nihlen, John Pernow, Mattias Carlström, Eddie Weitzberg, Richard Ågren, Jon O. Lundberg

**Affiliations:** a Department of Physiology and Pharmacology, Karolinska Institute, Stockholm, Sweden; b Department of Cell and Molecular Biology, Karolinska Institute, Stockholm, Sweden; c Department of Medicine, Karolinska Institutet, Karolinska University Hospital, Stockholm, Sweden; d Department of Evolutionary Genetics, Max Planck Institute for Evolutionary Anthropology, Leipzig, Germany; e Department of Cardiology, Karolinska University Hospital, Stockholm, Sweden

## Abstract

**Rationale:**

The entero-salivary circulation of inorganic nitrate (NO_3_^−^) involves the absorption of dietary nitrate in the gut and active uptake from blood by the salivary glands, leading to a 20-25-fold concentration in saliva. This recycling process is crucial for the nitrate-nitrite-nitric oxide (NO) pathway, which helps maintaining NO signaling in mammals. While the exact uptake mechanisms are unclear, sialin (encoded by the *SLC17A5* gene) has been suggested to play a key role. Interestingly, studies from the 1950s indicate that nitrate transport in the salivary glands competes with iodide (I^−^). This prompted us to explore the role of the sodium/iodide symporter (NIS) in salivary nitrate uptake.

**Method and results:**

Our database analysis revealed that the *SLC5A5* gene (encoding NIS) and its protein are expressed at higher levels than *SLC17A5* in the human salivary gland. Next, we expressed *SLC5A5* in *Xenopus Laevis* oocytes and its functionality using electrophysiology. We could detect ion influx induced by nitrate, indicating nitrate transport. We also observed increased levels of nitrate in *SLC5A5*-injected oocytes and in human salivary gland cells overexpressing *SLC5A5*, after incubation with nitrate. Finally, to test the competition between nitrate and iodide *in vivo*, saliva samples were collected from patients receiving high doses of intravenous iodine (I_2_) contrast medium, a procedure known to generate considerable levels of circulating I^−^. We observed a marked decrease in salivary nitrate following the administration of contrast medium, indicating competition for salivary transport.

**Conclusion:**

Our findings suggest that NIS is mediating salivary gland uptake and concentration of nitrate in saliva.

## 1 Introduction

The entero-salivary circulation of inorganic nitrate (NO_3_^−^) is a physiological process involving the absorption of dietary nitrate in the upper gastrointestinal tract, its active uptake by the salivary glands, and subsequent concentration in saliva. This recycling mechanism is a fundamental component of the nitrate-nitrite-nitric oxide (NO) pathway [1]. In this pathway, salivary nitrate is efficiently reduced to the more reactive anion nitrite (NO_2_^−^) by oral commensal bacteria, after which nitrite-rich saliva is swallowed and reaches the systemic circulation. In blood and tissues there are several mechanisms for further reduction of nitrite to NO and other bioactive nitrogen oxide species. This pathway functions in parallel to the classical NO synthase (NOS) system and is important for maintaining NO signaling in mammals [1]. Dietary nitrate is not the sole source of salivary nitrate since NOS-derived NO is oxidized to nitrate by blood hemoglobin and thereby inactivated [2]. The uptake of endogenously generated nitrate by the salivary glands describes a recycling of NO in the nitrate-nitrite-NO pathway. The recycling is a fundamental component of this pathway, which in addition to the classical NOS system is important for maintaining NO signaling in mammals. Interestingly, dietary nitrate has been shown to fuel this pathway, leading to positive effects on cardiovascular and metabolic health [[3], [4], [5]].

The significance of nitrate transport and concentration by the salivary glands is highlighted by their ability to sequester approximately 25 % of circulating nitrate, resulting in salivary nitrate levels that are about 20 times higher than those found in plasma [1,6]. Despite its importance, the specific mechanisms governing nitrate uptake, and the transporters involved in this process, remain only partially understood. Previous research has implicated sialin (encoded by the gene *SLC17A5)* as a key player and sialin is today considered to be the main salivary nitrate transporter [7]. At the same time, earlier studies have indicated that iodide (I^−^) uptake mediated by NIS in the thyroid occurs in competition with a number of other anions, including perchlorate (ClO_4_^−^), thiocyanate (SCN^−^), and nitrate, which is particularly interesting due to their dietary and environmental relevance [[8], [9], [10], [11], [12]]. These early observations together with the fact that active I^−^ transport takes place also in extrathyroidal tissues including the salivary glands [11], led us to hypothesize that NIS also facilitates active uptake of nitrate in the salivary glands.

## 2 Results

### 2.1 *SLC5A5* is highly expressed in human salivary glands

To investigate gene expression of *SLC5A*5 and *SLC17A5* in various human tissues, we first conducted an analysis across existing databases. The Human Protein Atlas tissue analysis (www.proteinatlas.org) revealed that *SLC5A5*, which encodes NIS, is most highly expressed in the choroid plexus, followed by the salivary glands, the stomach and the thyroid gland (Supplementary Table S1) [13,14]. In addition, similar results were observed in the FANTOM5 project database, which used Cap Analysis of Gene Expression (CAGE) data.

The highest expression of *SLC17A5,* which encodes for the proposed nitrate transporter sialin, is in the parathyroid gland, followed by the epididymis, thyroid gland, and kidney, with comparatively lower expression in salivary glands (Supplementary Table S2).

Overall, database analyses show higher SLC*5A5* expression in human salivary glands compared to *SLC17A5* (Supplementary Table S3).

### 2.2 Protein expression and location of NIS in the salivary gland

**Fig. 1:**
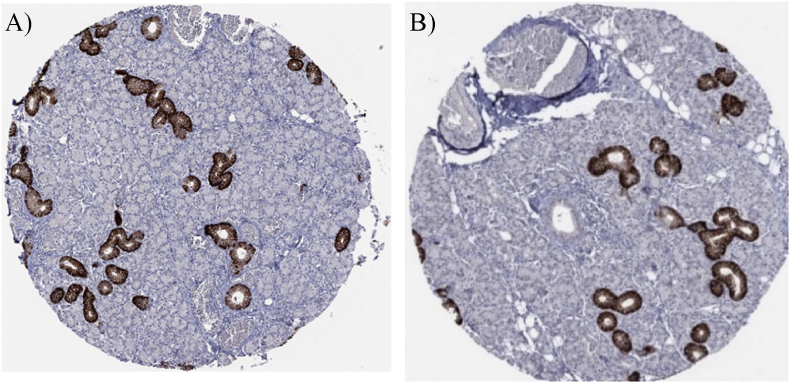
NIS is enriched in the salivary ducts. *NIS expression in human salivary glands detected by immunohistochemistry (IHC) with antibody staining (HPA049055) from the Human protein Atlas*. *A) Female, age 24: Salivary gland (T-55100) Normal tissue. B) Male, age 71: Salivary gland (T-55100), Normal tissue.*

Next, we used the Human Protein Atlas to examine NIS protein expression in the salivary glands. Notably, NIS is highly expressed at this site and exhibits histological dominance within the salivary ducts, more specifically in the intralobular salivary ducts (Fig. 1), characterized by a striated appearance due to membrane infoldings and aligned mitochondria [15]. These ducts are a part of the secretory lobules and are responsible for electrolyte uptake during both absorption and secretion processes (Supplementary Figure 1) [16]. Moreover, NIS is expressed at the basolateral plasma membrane of epithelial ductal cells, which would allow the involvement of NIS in uptake of nitrate in these cells. In contrast, there was no protein expression of sialin in the salivary gland in the Human Protein Atlas.

### 2.3 NIS promotes nitrate uptake *in vitro*

Previous studies of NIS suggest that nitrate uptake is electrogenic, with two sodium ions being co-transported with one nitrate ion [17,18], enabling the use of electrophysiology to study NIS-mediated transport using the Xenopus Expression System [7,8].

**Fig. 2:**
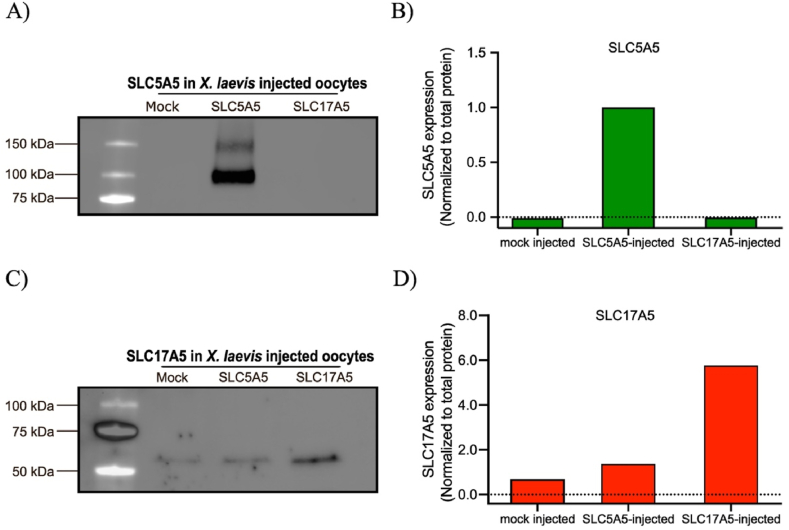
**Protein expression in Xenopus oocytes following mRNA injections.** A) Western blot of NIS expression. A distinct band at the expected molecular weight of NIS (100 kDa) was detected in the SLC5A5-injected oocyte lysate, while no signal was observed in mock- or SLC17A5-injected lysates. B) Quantification of the western blot in A). SLC5A5 band was normalized to total protein, expressed as fold change from lowest detected value for visualization purposes. C) Western blot of sialin expression. A prominent band at the expected molecular weight of sialin (55 kDa) was detected in the SLC17A5-injected oocyte lysate, whereas faint bands were observed in mock- and SLC5A5 lysates. D) Quantification of blot in C). SLC17A5 bands were normalized to total protein, expressed as fold change from lowest detected value for visualization purposes.

To study nitrate uptake, we microinjected human-derived *SLC5A5* and *SLC17A5* mRNA in stage V-VI *Xenopus Laevis* oocytes and confirmed successful heterologous protein expression via western blot analysis, which demonstrated robust overexpression of the NIS and sialin proteins, respectively (Fig. 2, Supplementary Fig. 2).

The expression pattern of *SLC17A5* aligns with its established role in encoding for the lysosomal transporter sialin, which is endogenously expressed in stage V–VI *X. laevis* oocytes (Supplementary Table S4) [19].

**Fig. 3:**
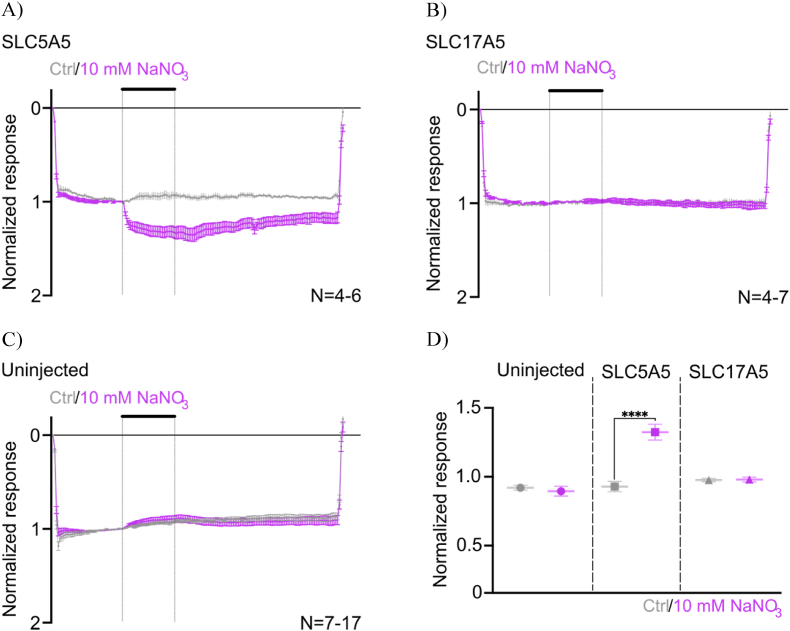
**Nitrate induces a putative sodium current in *SLC5A5*-expressing oocytes.***A) Application of* 10 mM *NaNO*_*3*_*solution (purple) evokes an inward current of* 5 nA *in SLC5A5-injected oocytes but not B) in SLC17A5-injected oocytes or C) in uninjected oocytes. Holding membrane potential of -*60 mV*. Recordings were performed at -*80 mV*. D) Mean currents (± SD) evoked by* 10 mM *NO*_*3*_^*−*^*in uninjected, SLC5A5 and SLC17A5 expressing oocytes at -*80 mV*. ∗∗∗∗: p < 0.0001.*

We then evaluated the transporters functionality using Two-Electrode Voltage-Clamp (TEVC). Perfusion with 10 mM NO_3_^−^ solution induced inward currents of ∼5 nA in *SLC5A5*-injected oocytes, indicating cation influx. Subsequent perfusion with the control solution did not elicit a current (Fig. 3A). In contrast, when perfusing with nitrate-rich solution uninjected and *SLC17A5*-injected oocytes we did not observe any current (Fig. 3B and C).

To further confirm the reliability of the currents associated with the nitrate-nitrite-NO pathway, we evaluated the transport activity of the two SLCs using a 10 mM nitrite (NO_2_^−^) solution. The nitrite perfusion did not induce any currents in the *SLC5A5* or *SLC17A5* injected oocytes (see Supplementary Figure 3) suggesting selectivity in transporter activity, in line with the nitrate-nitrite-NO recycling pathway, in which nitrite is not actively taken up by salivary glands.

Considering the variability in nitrate levels in human plasma and saliva, influenced by factors such as diet and endogenous NOS activity [3,16], we investigated the effects of physiologically relevant nitrate concentrations on *SLC5A5* and *SLC17A5* expressed in *X. laevis* oocytes. To do this, we incubated the oocytes in either nitrate-containing or control solution for 6 days, followed by quantification of the transported nitrate using High-Performance Liquid Chromatography (HPLC).

**Fig. 4:**
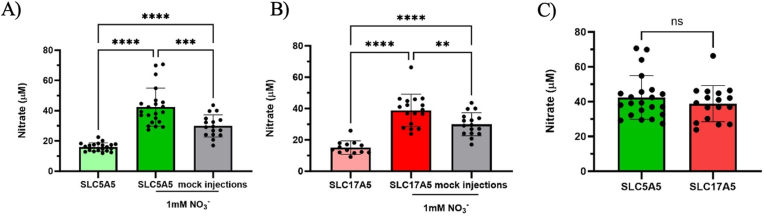
***SLC5A5*-injected oocytes accumulate nitrate.***Incubation with* 1 mM *NO*_*3*_^*−*^*resulted in increased intracellular nitrate concentrations in SLC5A5-injected oocytes (A) compared to controls as was the case with SLC17A5-injected oocytes (B). Both transporters also facilitated nitrate uptake relative to mock-injected oocytes (A, B). No significant difference in nitrate uptake was observed between SLC5A5 and SLC17A5 (C). ∗∗: p < 0.01, ∗∗∗: p < 0.001, ∗∗∗∗: p < 0.0001.*

We observed significant differences in nitrate uptake between oocytes exposed to 1 mM nitrate and those exposed to a control solution (Fig. 4A and B). Oocytes injected with *SLC5A5* showed approximately 2.6-fold higher intracellular nitrate than those exposed to control solution (42 ± 12 vs. 16 ± 3 μM); (Fig. 4A). Similarly, *SLC17A5*-injected oocytes exhibited roughly 2.5-fold higher intracellular nitrate levels when exposed to nitrate, reaching 39 ± 10 μM (Fig. 4B).

Mock injections confirmed that the observed nitrate uptake was specific to the *SLC5A5* and *SLC17A5* transporters. Accordingly, oocytes injected with *SLC5A5* RNA showed accumulated 1.4-fold more nitrate compared to mock-injected oocytes (42 ± 12 vs. 30 ± 7 μM); (Fig. 4A).

Similarly, oocytes injected with *SLC17A5* RNA exhibited 1.3-fold higher nitrate compared to mock-injected (39 ± 10 vs. 30 ± 7 μM); (Fig. 4B). The comparison of nitrate uptake between *SLC5A5* and *SLC17A5* revealed no significant difference (p = 0.35) (Fig. 4C).

Together, these results demonstrate that *SLC5A5* mediates electrogenic nitrate transport in *X. laevis* oocytes and that prolonged exposure enhances intracellular nitrate accumulation in both *SLC5A5*-and *SLC17A5*-expressing oocytes.

As mentioned above, *X. laevis* oocytes provide a valuable model for studying NIS-mediated transport using TEVC. However, nitrate accumulation experiments in this system require prolonged incubation periods (6 days), which differ considerably from salivary transport kinetics observed in humans, where salivary nitrate rises rapidly following dietary intake, reaching elevated levels already within 15 minutes [20,21], highlighting a physiologically relevant window for rapid nitrate uptake.

To determine whether NIS mediates nitrate uptake also in human salivary epithelial cells, we overexpressed SLC5A5 in A-253 cells and exposed them to nitrate-containing media, followed by quantification of intracellular nitrate by HPLC. In the first set of experiments, we incubated cells for 5 min in complete growth medium supplemented with 1 mM NaNO_3_, corresponding to plasma nitrate levels observed after dietary nitrate load (e.g., drinking beetroot juice) [22].

**Fig. 5:**
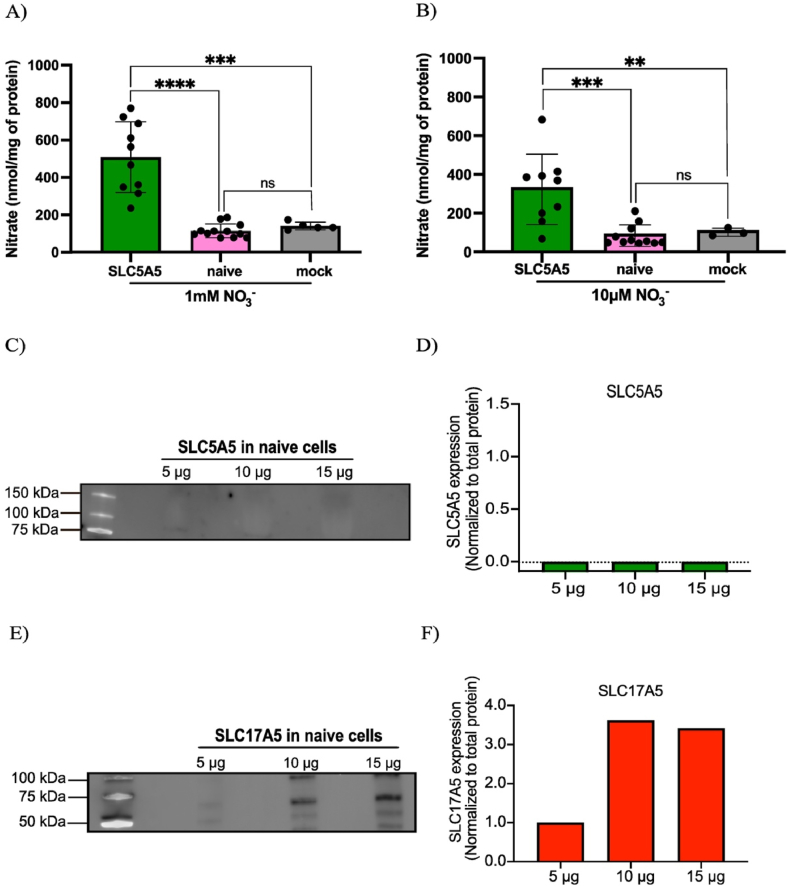
**Nitrate uptake in A-253 cells is enhanced by SLC5A5 overexpression.***A) Acute exposure to* 1 mM *NO*_*3*_^*−*^*led to an increase in intracellular nitrate in SLC5A5-transfected cells compared to naive cells mock-transfected cells, with no significant difference between naive and mock-transfected cells. B) After 24 h in low-nitrate medium (*10 μM *NO*_*3*_^*−*^*), SLC5A5-transfected cells accumulated more nitrate than naive cells mock-transfected cells, with no difference between naive and mock-transfected cells. C) Western blot of NIS (SLC5A5) expression in naive A-253 cells. No detectable band was observed at the expected molecular weight across three different protein loads. D) Quantification of the blot in C. The SLC5A5 signal was normalized to total protein and expressed as fold change from the lowest detected value for visualization purposes. E) Western blot of sialin (SLC17A5) expression in naive A-253 cells. A prominent band at the expected molecular weight of SLC17A5 (*55 kDa*) was observed across three different protein loads. F) Quantification of the blot in E. SLC17A5 signal was normalized to total protein and expressed as fold change from the lowest detected value for visualization purposes. ∗∗: p < 0.01, ∗∗∗: p < 0.001, ∗∗∗∗: p < 0.0001.*

*SLC5A5*-overexpressing cells accumulated significantly more nitrate than naive cells (508.6 ± 189.0 vs. 125.3 ± 34.8 nmol/mg protein) and mock-transfected cells (140.4 ± 20.8 nmol/mg protein), corresponding to approximately 4-fold and 3.6-fold higher nitrate levels, respectively (Fig. 5A).

Under low-nitrate conditions (10 μM NO_3_^−^) for 24 hours, approximating fasting plasma levels more closely [22], *SLC5A5*-overexpressing cells again showed significantly higher nitrate levels than naive cells (325.1 ± 181.4 vs. 81.4 ± 48.4 nmol/mg protein) and mock-transfected cells (105 ± 21 nmol/mg protein), corresponding to approximately 4-fold and 3-fold higher levels, respectively (Fig. 5B).

In parallel, we also examined basal expression of putative nitrate transporters to assess the potential contribution of endogenous transporters in A-253 cells. Interestingly, western blot analysis revealed that while NIS protein (*SLC5A5*) was not detectable in naive cells, sialin (*SLC17A5*) was expressed (Fig. 5C–F, Supplementary Fig. 5). This endogenous sialin expression aligns with its primary role as a lysosomal transporter, which is broadly expressed in mammalian cells [23]. The lack of detectable NIS expression and the absence of significant nitrate uptake upon nitrate exposure in naive cells indicate that sialin alone supports only limited intracellular nitrate accumulation in A-253 cells. This finding is consistent with the carcinoma origin of the A-253 cell line, as NIS expression in salivary glands is decreased during inflammation and tumorigenesis [24].

Together, these findings show that nitrate uptake in *SLC5A5*-expressing cells was markedly higher than in naive and mock-transfected controls, supporting a role for NIS as the primary mediator of intracellular nitrate uptake in A-253 cells.

### 2.4 Iodine-containing contrast medium reduces salivary nitrate levels *in humans*

To evaluate a possible competition between nitrate and iodide in an *in vivo* setting, we next investigated the impact of iodine-containing contrast medium on salivary nitrate levels in patients undergoing coronary angiography or Percutaneous Coronary Intervention (PCI). The mean volume of contrast medium (Iomeron) given to the patients was 176 ± 116 ml and varied depending on body weight and complexity of the procedure (Supplementary Table S5).

**Fig. 6:**
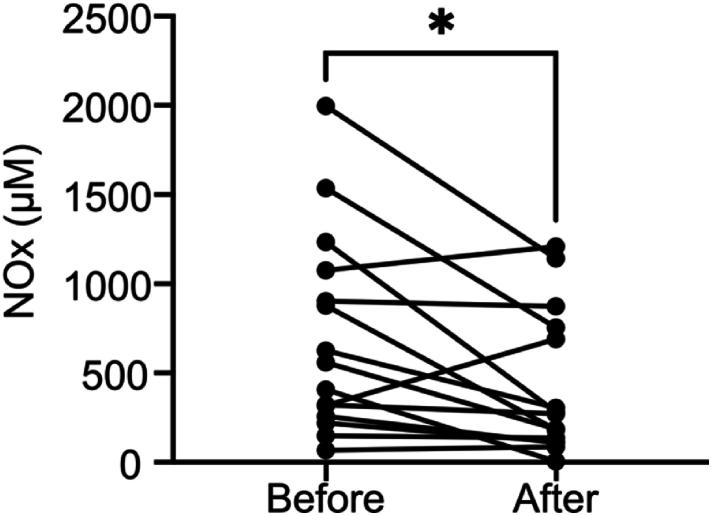
**Salivary nitrate levels decrease after administration of iodine-containing contrast medium.***Data from 15 patients receiving Iomeron contrast medium (*350 mg *I*_*2*_*/mL) demonstrate a significant reduction in saliva NOx concentration (p < 0.05, ∗) following its administration. This finding suggests a competitive interaction between iodide (I*^*−*^*) and nitrate (NO*_*3*_^*−*^*) for salivary transport.*

Our measurements revealed that the average basal salivary nitrate + nitrite (NOx) concentration decreased significantly from 704 ± 561 μM before administration to 423 ± 401 μM (p = 0.015) after the administration of iodine-containing contrast medium (Fig. 6). These results clearly indicate a competition *in vivo* between iodide and nitrate for transport in the salivary glands, which supports the notion that nitrate is utilising NIS for its transport between blood and saliva. Since salivary nitrite is solely derived from nitrate secreted by the salivary glands after being reduced by oral bacteria, we report the combined nitrate + nitrite (NOx) concentration.

## 3 Methods

**Ethical approval**: The study in humans was approved by the Ethics Review Authority in Sweden.

### 3.1 Database analysis

To assess the expression levels of *SLC5A5* gene (NIS) and *SLC17A5* gene (sialin), we analyzed data from the HPA, FANTOM5 CAGE and GTEx projects [13,25,26]. We used histological information from the HPA to further investigate NIS protein expression and localization in human salivary glands. For additional insights, we incorporated data from the Expression Atlas for *Mus musculus*.

### 3.2 Nitrate transport in cells

We studied the functional properties of SLC transporters using the *X. laevis* oocytes as an expression system, which is ideal for examining the relationship between the transporters' electrical properties and their solute translocation abilities [27].

### 3.3 Oocytes preparation and protein expression

We retrieved oocytes from Ecocyte Biosciences (Dortmund, Germany). We started by microinjecting 40 ng/oocyte of *SLC5A5* and *SLC17A5* cRNA into stage V-VI *X. laevis* oocytes using the Nanoject III (Drummond Scientific). We maintained the oocytes in Modified Barth's Solution (MBS) at 12 °C until use. After injection, we incubated the oocytes for 6–7 days at 14 ± 2 °C in fresh oocyte culture medium (MBS: 88 mM NaCl, 1 mM KCl, 0.82 mM MgSO_4_, 0.33 mM Ca(NO_3_)_2_, 0.41 mM CaCl_2_, 10 mM HEPES, 25 U/mL penicillin, and 25 μg/mL streptomycin, adjusted to pH 7.4 with NaOH).

### 3.4 Electrophysiological measurements

After incubation, we performed Two-Electrode Voltage-Clamp (TEVC) recordings at room temperature (RT, 22 °C) with the OpusXpress, a semi-automated system for high throughput voltage clamp recording [28]. During recordings, we perfused the oocytes with control solution containing: 100 mM NaCl, 2 mM KCl, 1 mM CaCl_2_, 1 mM MgCl_2_, and 10 mM HEPES, (adjusted to pH 7.4 using NaOH). For electrophysiology experiments, we replaced NaCl with NaNO_3_, retaining the total Na ^+^ concentration. We evoked currents in *SLC5A5*-and *SLC17A5*-injected oocytes by voltage-clamping at a holding potential of −60 mV and stepping down to −80 mV to establish a baseline prior to applications. We continuously perfused to exchange the background buffer for 10 mM NaNO_3_ or NaNO_2_.

### 3.5 Western blotting of X. Laevis oocytes

One week after mRNA microinjection, we isolated the proteins from Xenopus oocytes, following a protocol adapted from Vleminckx and colleagues [29]. The oocytes were lysed in a buffer containing 1 % Igepal, 25 mM sucrose, 10 mM EDTA, 25 mM HEPES, and a Protease & Phosphatase Inhibitor Cocktail 100X (Thermo Scientific, #78442) at a 1:100 dilution. Then, the cells were homogenized by pipetting up and down 10 times, incubated at 37 °C for 10 min, and vortexed for 10 s. After centrifugation at 2500 rcf, we collected the middle aqueous phase containing proteins, leaving behind the top lipid layer and the pellet containing the yolk platelets. Protein concentrations were determined using the Pierce™ BCA Protein Assay (Thermo Scientific, #23227).

For protein separation, we mixed 10 μg from each protein sample with 4x Laemmli buffer (BioRad, #1610747) supplemented with 5 % 2-mercaptoethanol (BioRad, #1610710) in a 0.5 mL tube. Samples were denatured at 95 °C for 5 min, followed by centrifugation at 20,817 rcf for 1 min. Electrophoresis was performed using a Mini-PROTEAN TGX Stain-Free Gel (BioRad, #4568094). We prepared buffer by diluting 10x TGS stock solution (BioRad, #1610772) in MQH_2_O (900 mL MQH_2_O + 100 mL 10x TGS). We loaded protein samples and 3 μL of Precision Plus Protein™ All Blue Prestained Protein Standards (BioRad, #1610373) onto the gel and separated at 200 V for 40 min using a PowerPac™ Basic power supply (BioRad, #1645050). Following electrophoresis, we used stain-free imaging technology to activate the gel (ChemiDoc™ MP Imaging System, BioRad, #12003154). We activated a low-fluorescence PVDF membrane in methanol and prepared filter papers soaked in transfer buffer (Trans-Blot Turbo RTA Transfer Kit LF PVDF, BioRad, #1704274) for blotting. We transferred the proteins using a Trans-Blot Turbo Transfer System (BioRad, #1704150) under the mixed molecular weight program for 7 min. We verified successful transfer by imaging total protein on the membrane using stain-free technology.

We blocked the membranes in EveryBlot Blocking Buffer (BioRad, #12010020) for 10 min at RT on a shaker. After blocking, we incubated the membranes overnight at 4 °C with either SLC5A5 (Sigma, #MAB3564, 1:200) or SLC17A5 (Abcam, #ab153920, 1:100) primary antibody, both diluted in 5 % skim milk (Millipore, #70166-500G). We washed the membranes three times for 10 min in TBST (ChemCruz, #SC-362311) and incubated them for 1 h at RT on a shaker with either Anti-Mouse IgG Peroxidase (Abcam, #ab205719, 1:5000) or Anti-Rabbit IgG Peroxidase (Abcam, #ab206722, 1:5000), both diluted in 5 % skim milk. After incubation, we washed the membranes twice for 10 min in TBST and once for 10 min in PBS. Then, we incubated the membranes for 5 min at RT in 5 mL of SuperSignal™ West Pico PLUS Chemiluminescent Substrate (Thermo Scientific, #34580). We performed the imaging on a ChemiDoc™ MP Imaging System, and quantified protein bands using ImageLab (BioRad).

### 3.6 Nitrate accumulation assay in X. Laevis oocytes

The use of *X. laevis* oocytes as a heterologous host for the expression of secondary active transporters extends beyond electrophysiology. We also performed uptake assays followed by detection and quantification of intracellular nitrate.

We began by incubating *SLC5A5-*, *SLC17A5-*and mock-injected oocytes in either nitrate-free MBS or 1 mM NaNO_3_ MBS for 6 days at 12 °C. After two washout steps, we transferred three oocytes into an Eppendorf tube, carefully removed the external solution, homogenized the oocytes, and added methanol. We then centrifuged the samples for 5 min at a fixed speed of 6000 min^−1^ (2000×*g*) using a MiniStar centrifuge and sampled approximately 25 μL of the supernatant and snap-frozen at −80 °C until use.

### 3.7 Nitrate quantification by High-Performance Liquid Chromatography (HPLC)

We quantified nitrate and nitrite levels using a sensitive HPLC method based on ion exchange chromatography, as described in previous studies [6]. We calibrated the system with sodium nitrate and nitrite standards (0.1–20 μM), and 10 μL of each sample was injected for analysis. It is known that pure ductal secretions of saliva contain only nitrate not nitrite. Once in the oral cavity parts of the nitrate are reduced to nitrite by commensal oral bacteria [1]. Because the degree of nitrate reduction to nitrite can vary greatly between individuals, the combined measurement of nitrate + nitrite gives a better estimate of true nitrate secretion in saliva which is what is being studied here. Therefore, in the human study nitrate values displayed for saliva represent nitrate + nitrite, sometimes referred to as NOx. A similar pattern was seen also if displaying nitrate and nitrite separately (not shown).

### 3.8 Culture of A-253 cells

The human submandibular gland carcinoma cell line A-253 (ATCC® HTB-41™) served as a model of salivary gland epithelial cells. Cells grew in McCoy's 5A Modified medium (Gibco, Thermo Fisher Scientific catalog #16600082 or Cytiva, catalog # SH30200.01) supplemented with 10 % (v/v) heat-inactivated fetal bovine serum (FBS; Gibco, Thermo Fisher Scientific catalog #A5670801) and 1 % penicillin-streptomycin (100 U/mL penicillin, 100 μg/mL streptomycin) at 37 °C in a humidified incubator with 5 % CO_2_. We routinely passaged cells at 70–90 % confluence using 0.25 % trypsin-EDTA (catalog #25200056). We performed all experiments using cells between passages 3 and 6.

### 3.9 Transient transfection of A-253 cells

We performed transient transfections using Lipofectamine™ 3000 (Invitrogen, Thermo Fisher Scientific) according to the manufacturer's protocol with minor optimization. A-253 cells reached 80–90 % confluence in 12-well plates before transfection. We transfected cells with either pcDNA3.1-eGFP-SLC5A5 or empty pcDNA3.1-eGFP vector as mock control (all custom-made and purchased from GenScript). For each well, we diluted 0.5 μg plasmid in 125 μL FBS-free McCoy's 5A Modified medium (Thermo Fisher Scientific catalog #16600082, or Cytiva, catalog # SH30200.01) supplemented with 1 % penicillin-streptomycin, together with 1 μL P3000™ reagent. Separately, we diluted 1 μL Lipofectamine™ 3000 in equal volume of FBS-free medium and vortexed for 2–3 s. We combined the DNA/P3000 mixture and the Lipofectamine solution and incubated it for 15 min at room temperature to allow lipid-DNA complex formation before addition to cells. We added the resulting transfection mixture was added to complete growth medium (McCoy's 5A Modified medium supplemented with 10 % FBS and 1 % penicillin-streptomycin) and cultured the cells for 24 h prior to imaging and nitrate uptake assay.

### 3.10 Brightfield and fluorescence microscopy of A-253 transfected cells

We used brightfield and fluorescence microscopy on an EVOS M7000 Imaging System (Thermo Fisher Scientific) with a 10 × objective to assess transfection efficiency and cell confluence. Microscopy 24 h after transfection in fresh growth medium confirmed that fluorescence originated from viable, adherent cells rather than debris or detached cells. We analyzed images of eGFP expression qualitatively using in-house semi-automated macros developed in Fiji (ImageJ). Representative images appear in Supplementary Figure 4.

### 3.11 Nitrate uptake assay in A-253 cells

To develop the nitrate uptake assay, we adapted and combined approaches from previous studies. Specifically, we based our short-term, non-radioactive uptake protocol on the method described by Song et al. for solute carrier transporters, and incorporated aspects of nitrate handling from the protocol used by Srihirun et al. in human skeletal muscle cell cultures [30,31]. This approach allowed us to measure intracellular nitrate accumulation in A-253 cells, with normalization to total protein content to account for differences in cell number and lysis efficiency.

### 3.12 Acute high-nitrate exposure: mimicking post-dietary plasma levels

After confirming cell confluence and transfection efficiency with brightfield and fluorescence microscopy in fresh complete growth medium to ensure that the signal originated from viable cells rather than debris or detached cells, we then incubated cells in complete growth medium supplemented with 1 mM NaNO_3_ for 5 min. Following incubation, we rinsed cells with cold PBS and lysed in Milli-Q water containing 1 % Sodium Dodecyl Sulfate (SDS) (catalog #28312) and Halt™ Protease and Phosphatase Inhibitor Cocktail, EDTA-free X100 at a 1:100 dilution (Thermo Scientific, catalog #78441 or 2).

We clarified lysates by centrifugation at 20,000×*g* for 10 min at 4 °C and discarded any precipitated chromatin-like material. From the clarified lysate, we used 10 μL to determine total protein concentration using the Pierce™ BCA Protein Assay Kit (catalog #23227), which employs a colorimetric method based on the reduction of Cu^2+^ to Cu ^+^ by protein in an alkaline medium, followed by detection using bicinchoninic acid. We diluted the remaining lysate 1:1 with ice-cold methanol and stored it at −20 °C until nitrate quantification by HPLC.

### 3.13 Low-nitrate exposure: mimicking fasting plasma levels

All growth media, including both FBS-free and complete serum-containing formulations, contain residual nitrate (10–500 μM unpublished observations), making completely nitrate-free conditions not achievable. Instead, we decided to take advantage of the intrinsic nitrate already present under basal conditions for a long-term low-nitrate exposure experiment. In fact, 10 μM resembles well fasting plasma nitrate levels in humans. Thus, A-253 cells were maintained in low-nitrate medium (10 μM NO_3_^−^, measured with HPLC) for 24 h, during the transfection period. Following incubation, we assessed cell confluence and transfection efficiency using brightfield and fluorescence microscopy in fresh complete growth medium. We then processed the cells for protein quantification and intracellular nitrate measurement using the same procedures as described for the acute high-nitrate condition.

### 3.14 Protein quantification

We quantified total protein using the Pierce™ BCA Protein Assay Kit (catalog #23227) in 96-well plates with a Bovine Serum Albumin (BSA) standard curve (0–2 mg/mL, in duplicates). Samples (10 μL, in duplicates) mixed with 200 μL BCA solution and incubated at 37 °C for 30 min before measuring absorbance at 562 nm. Measured protein concentrations were then used to normalize intracellular nitrate levels.

### 3.15 Saliva samples from patients receiving iodine-containing contrast medium

To investigate a possible transport competition between nitrate and iodide (I^−^) *in vivo*, we collected saliva samples from 15 patients with chronic coronary syndrome undergoing coronary angiography or PCI and receiving Iomeron contrast medium (Bracco, Sweden, 350 mg I/ml). The patients arrived at the hospital in the morning after a light breakfast. We collected saliva samples before and after (2–6 h) the administration of iodinated-contrast medium, then stored frozen (−80 °C) and analyzed for nitrate levels (using the HPLC system described above).

### 3.16 Data presentation and statistical analysis

We used PowerChrom software to process HPLC data and pClamp and Clampfit software analyzed electrophysiological data obtained via TEVC. We filtered the currents using a 3 Hz low-pass filter and smoothened by applying a 500-point (3.2 s) moving average. We performed statistical analysis and graphical representations for both datasets using GraphPad Prism version 9.2.0 (GraphPad Software).

We present HPLC data as mean ± standard deviation (SD) and TEVC data as mean ± standard error of the mean (SEM), unless stated otherwise. TEVC data are expressed as mean ± standard error of the mean (SEM), if not specified differently. For each oocyte in the TEVC experiments, we normalized the current response after 60 s of nitrate or nitrite application to current preceding the application in the same oocyte.

We performed stain-free imaging of membranes using a ChemiDoc™ MP Imaging System (BioRad). We quantified protein bands using ImageLab software (BioRad), with total protein densitometry determined via the ImageLab volume tool. To account for loading differences, we normalized the signal intensities of proteins of interest to the total protein content of the corresponding well.

We asses data distribution using the Shapiro–Wilk test for normality. For normally distributed data with equal variances, we used ordinary one-way ANOVA or unpaired t-tests assuming equal SD. If normality or equal variance assumptions were not met, we applied appropriate non-parametric tests or t-tests not assuming equal SD. Statistical significance was defined as p < 0.05. Statistical analyses were done using GraphPad Prism® version 10.6.0.

## 4 Discussion

The data presented here suggest that the sodium/iodide symporter (NIS) mediates both rapid and prolonged uptake of circulating inorganic nitrate in the salivary glands. NIS is highly expressed in human salivary tissue, and *SLC5A5*-injected oocytes readily transport nitrate. Consistently, overexpression of *SLC5A5* in human A-253 salivary epithelial cells promotes nitrate uptake. Moreover, our human *in vivo* data indicate competition between iodide and nitrate for salivary transport. Together, these findings support a role of NIS in mediating physiological nitrate transport in human salivary glands.

The entero-salivary circulation of nitrate (NO_3_^−^) is a crucial physiological process that involves the absorption of dietary nitrate in the upper gastrointestinal tract, its active uptake by the salivary glands, and subsequent concentration in saliva. This recycling mechanism is fundamental to supporting the nitrate-nitrite-nitric oxide (NO) pathway, which complements the classical nitric oxide synthase (NOS) system in generating bioactive NO species [1]. Central to this pathway is the reduction of nitrate to nitrite by commensal oral bacteria, a process sustained by the entero-salivary circulation.

The significance of this circulation is underscored by the fact that approximately 25 % of circulating nitrate is sequestered by the salivary glands, leading to salivary nitrate levels that are 20 times higher than those found in plasma. This accumulation is a prerequisite for a functional nitrate-nitrite-NO pathway. Previous studies identified sialin (encoded by *SLC17A5*) as the primary nitrate transporter [7], meanwhile our findings suggest that NIS plays a significant role in nitrate uptake and concentration in human saliva.

Our results do not directly refute sialin as an additional nitrate transporter since we did note nitrate accumulation in the *X. laevis* oocytes injected with *SLC17A5* mRNA after prolonged exposure to nitrate-rich medium, suggesting a potential complementary role of sialin and NIS in slow nitrate transport. The overall nitrate transport system in the human salivary gland might reflect a low-affinity, high-capacity system. Such coexistence of rapid and slower nitrate transport components is analogous to bacteria, which commonly possess both low-affinity (fast) and high-affinity (slow) nitrate transporters to function across a wide range of nitrate concentrations [20 32]. Nevertheless, altogether our findings suggest a quantitatively more significant role for NIS in salivary nitrate transport and in particular the rapid uptake as seen after dietary exposure to nitrate [30]. This conclusion is reinforced by Human Protein Atlas database analysis, electrophysiological findings supported by western blot validation of robust NIS and sialin overexpression in oocytes, ensuring that observed transport differences reflect intrinsic transporter function rather than protein expression variability. The consistent findings across oocytes, human A-253 salivary epithelial cells, and *in vivo* data from patients collectively support a role of NIS in mediating salivary nitrate transport.

Interestingly, previous findings from our group show that rodents are less capable of concentrating nitrate in their saliva compared to humans [6]. When analyzing the Expression Atlas dataset, we found that in *Mus musculus*, S*lc5a5* expression in the salivary glands is approximately 16-fold lower than that of *Slc17a5* (Supplementary Table S6.). This reverse expression of the gene transporters in mice compared to humans coupled to reduced ability to concentrate nitrate in rodents further supports a role for NIS in human salivary gland uptake of nitrate and a lesser importance of sialin.

Studies from the 1950s have indicated a potential competition between iodide and nitrate for salivary transport [8] but surprisingly this has never been studied in the context of the nitrate-nitrite-NO pathway. To assess a putative interaction between nitrate and iodide, we examined the effects of iodine-containing contrast medium on salivary nitrate levels in patients receiving iodine-based contrast media as part of undergoing coronary angiography. Iodine-based contrast agents, such as Iomeron, are known to significantly increase free iodide (I^−^) during clinical use [25,26,33,34]. In line with our hypothesis, nitrate levels were significantly reduced in saliva samples of patients receiving iodinated contrast medium. This observation strongly supports the notion that a competitive interaction exists between nitrate and iodide for transport via NIS [35]. Collectively, our findings indicate that NIS plays an important role in nitrate transport and concentration within human salivary glands *in vivo*.

In conclusion, our results identify NIS as a physiologically relevant transporter of nitrate in human salivary glands, mediating both rapid and sustained intracellular transport. This study broadens our knowledge of nitrate transport mechanisms in the entero-salivary circulation, emphasizing the relationship between dietary nitrate intake, salivary gland function, and systemic NO bioactivity. Future research should explore clinical implications of altered nitrate transport dynamics and investigate therapeutic strategies targeting NIS.

## CRediT authorship contribution statement

**Gaia Picozzi:** Conceptualization, Formal analysis, Investigation, Methodology, Writing – original draft, Writing – review & editing. **Leo J.S. Westerberg:** Formal analysis, Investigation, Methodology, Writing – review & editing. **Juliane Jurga:** Investigation, Writing – review & editing. **Hugo Zeberg:** Formal analysis, Supervision, Writing – review & editing. **Carina Nihlen:** Methodology, Writing – review & editing. **John Pernow:** Investigation, Resources, Writing – review & editing. **Mattias Carlström:** Investigation, Methodology, Supervision, Writing – original draft, Writing – review & editing. **Eddie Weitzberg:** Conceptualization, Formal analysis, Investigation, Supervision, Writing – original draft, Writing – review & editing. **Richard Ågren:** Conceptualization, Supervision, Writing – original draft, Writing – review & editing. **Jon O. Lundberg:** Conceptualization, Formal analysis, Methodology, Supervision, Writing – original draft, Writing – review & editing.

## Declaration of competing interest

JOL and EW are co-inventors of patents describing the medical uses of inorganic nitrate and nitrite and co-directors of Heartbeet Ltd. The rest of the authors report no conflict of interest. All authors declare that they have no personal relationships that could have appeared to influence the work reported in this paper.
