# Supplementary material for: The sodium/iodide symporter is a nitrate transporter in the human salivary gland
Source: Redox Biol. 2025 Nov 29;89:103955. doi: 10.1016/j.redox.2025.103955 (PMC12721291; doi:10.1016/j.redox.2025.103955)
Supplement: Multimedia component 1 [file mmc1.docx]

***Supplementary Information***

***The sodium/iodide symporter is a nitrate transporter in the human salivary gland***

Gaia Picozzi^1^, Leo J.S. Westerberg^2^, Juliane Jurga^3^, Hugo Zeberg^1,4^, Carina Nihlen^1^, John Pernow^5^, Mattias Carlström^1^, Eddie Weitzberg^1^, Richard Ågren^1*^, Jon O. Lundberg^1*^

1. Department of Physiology and Pharmacology, Karolinska Institute, Stockholm, Sweden.
2. Department of Cell and Molecular Biology, Karolinska Institute, Stockholm, Sweden.
3. Department of Medicine, Karolinska Institutet, Karolinska University Hospital, Stockholm, Sweden.
4. Department of Evolutionary Genetics, Max Planck Institute for Evolutionary Anthropology, Leipzig, Germany.
5. Department of Cardiology, Karolinska University Hospital, Stockholm, Sweden.

*Shared senior authors

**Supplementary Table** **S1**. *Expression of SLC5A5 in 53 tissues based on transcriptomics data from the Human Protein Atlas (proteinatlas.org).*

| **Gene name** | **Tissue** | **nTPM** |
| --- | --- | --- |
| *SLC5A5* | choroid plexus | 163,3 |
| ***SLC5A5*** | **salivary gland** | **64,4** |
| *SLC5A5* | stomach | 58,8 |
| *SLC5A5* | thyroid gland | 12 |
| *SLC5A5* | hippocampal formation | 10,6 |
| *SLC5A5* | cerebellum | 5,6 |
| *SLC5A5* | cervix | 5,6 |
| *SLC5A5* | thalamus | 3,3 |
| *SLC5A5* | cerebral cortex | 1,4 |
| *SLC5A5* | midbrain | 0,7 |
| *SLC5A5* | pons | 0,4 |
| *SLC5A5* | skeletal muscle | 0,4 |
| *SLC5A5* | testis | 0,4 |
| *SLC5A5* | amygdala | 0,3 |
| *SLC5A5* | bone marrow | 0,3 |
| *SLC5A5* | lymph node | 0,3 |
| *SLC5A5* | pituitary gland | 0,3 |
| *SLC5A5* | white matter | 0,3 |
| *SLC5A5* | appendix | 0,2 |
| *SLC5A5* | endometrium | 0,2 |
| *SLC5A5* | medulla oblongata | 0,2 |
| *SLC5A5* | skin | 0,2 |
| *SLC5A5* | small intestine | 0,2 |
| *SLC5A5* | spleen | 0,2 |
| *SLC5A5* | urinary bladder | 0,2 |
| *SLC5A5* | basal ganglia | 0,1 |
| *SLC5A5* | breast | 0,1 |
| *SLC5A5* | esophagus | 0,1 |
| *SLC5A5* | gallbladder | 0,1 |
| *SLC5A5* | hypothalamus | 0,1 |
| *SLC5A5* | lung | 0,1 |
| *SLC5A5* | prostate | 0,1 |
| *SLC5A5* | retina | 0,1 |
| *SLC5A5* | spinal cord | 0,1 |
| *SLC5A5* | tonsil | 0,1 |
| *SLC5A5* | adipose tissue | 0 |
| *SLC5A5* | adrenal gland | 0 |
| *SLC5A5* | colon | 0 |
| *SLC5A5* | duodenum | 0 |
| *SLC5A5* | epididymis | 0 |
| *SLC5A5* | fallopian tube | 0 |
| *SLC5A5* | heart muscle | 0 |
| *SLC5A5* | kidney | 0 |
| *SLC5A5* | liver | 0 |
| *SLC5A5* | ovary | 0 |
| *SLC5A5* | pancreas | 0 |
| *SLC5A5* | parathyroid gland | 0 |
| *SLC5A5* | placenta | 0 |
| *SLC5A5* | rectum | 0 |
| *SLC5A5* | seminal vesicle | 0 |
| *SLC5A5* | smooth muscle | 0 |
| *SLC5A5* | thymus | 0 |
| *SLC5A5* | tongue | 0 |
| *SLC5A5* | vagina | 0 |

**Supplementary Table S2**. *Expression of SLC17A5 in 39 tissues based on transcriptomics data from the Human Protein Atlas (proteinatlas.org).*

| Gene name | Tissue | nTPM |
| --- | --- | --- |
| *SLC17A5* | parathyroid gland | 300,3 |
| *SLC17A5* | epididymis | 43,5 |
| *SLC17A5* | thyroid gland | 42,2 |
| *SLC17A5* | kidney | 30,7 |
| *SLC17A5* | **salivary gland** | **29,4** |
| *SLC17A5* | rectum | 21,4 |
| *SLC17A5* | placenta | 21 |
| *SLC17A5* | small intestine | 20,5 |
| *SLC17A5* | colon | 19,7 |
| *SLC17A5* | liver | 17,1 |
| *SLC17A5* | prostate | 16,2 |
| *SLC17A5* | duodenum | 16 |
| *SLC17A5* | seminal vesicle | 14,2 |
| *SLC17A5* | stomach | 14 |
| *SLC17A5* | breast | 13,3 |
| *SLC17A5* | lung | 12,8 |
| *SLC17A5* | urinary bladder | 12,1 |
| *SLC17A5* | pancreas | 11,3 |
| *SLC17A5* | cervix | 10,3 |
| *SLC17A5* | adrenal gland | 10,2 |
| *SLC17A5* | smooth muscle | 10,2 |
| *SLC17A5* | gallbladder | 10,1 |
| *SLC17A5* | adipose tissue | 9,9 |
| *SLC17A5* | endometrium | 9,1 |
| *SLC17A5* | esophagus | 8,1 |
| *SLC17A5* | heart muscle | 7,4 |
| *SLC17A5* | skin | 6,9 |
| *SLC17A5* | spleen | 6,8 |
| *SLC17A5* | bone marrow | 6,7 |
| *SLC17A5* | cerebral cortex | 6,7 |
| *SLC17A5* | appendix | 6,2 |
| *SLC17A5* | testis | 6 |
| *SLC17A5* | tongue | 6 |
| *SLC17A5* | choroid plexus | 5,7 |
| *SLC17A5* | tonsil | 5,4 |
| *SLC17A5* | lymph node | 5,2 |
| *SLC17A5* | fallopian tube | 5,1 |
| *SLC17A5* | thymus | 4,4 |
| *SLC17A5* | skeletal muscle | 4,3 |
| *SLC17A5* | ovary | 3,9 |

**Supplementary Table S3.** *Comparison between SLC5A5 and SLC17A5 in human salivary gland tissue based on transcriptomics data*

| Database | Gene | Species | Level of expression | Average | u.m. |
| --- | --- | --- | --- | --- | --- |
| HPA | *SLC5A5* | human | 64,4 | 33,9 | nTPM |
| GTEx | *SLC5A5* | human | 3,4 |  | nTPM |
| FANTOM5 CAGE | *SLC5A5* | human | 262,5 | - | Scaled Per million |
|  |  |  |  |  |  |
| HPA | *SLC17A5* | human | 29,4 | 24,35 | nTPM |
| GTEx | *SLC17A5* | human | 19,3 |  | nTPM |
| FANTOM5 CAGE | *SLC17A5* | human | 43,4 | - | Scaled Per Million |

Human Protein Atlas: HPA, GTEx: Genotype-Tissue Expression project, FANTOM5: Functional Annotation of Mammalian Genomes 5 project, CAGE: Cap Analysis of Gene Expression, nTPM: normalized Transcript Per Million, TPM: Transcript Per Million.


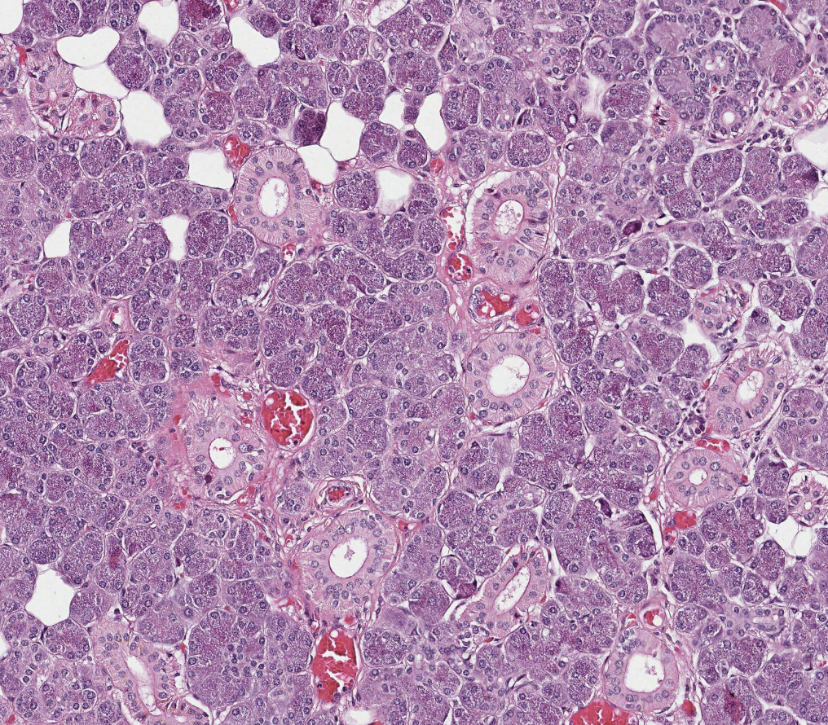
**Supplementary Figure 1. Normal salivary gland tissue.** *Section of salivary gland stained with hematoxylin and eosin for contrast from HPA, illustrating intralobular ducts along with supporting capillaries.*

**Supplementary Figure 2.** *Uncropped total protein and protein stains from* ***Figure 2****. A) Stain-free image of total protein used for normalization of NIS staining. B) NIS protein stain. C) Stain-free image of total protein used for normalization of Sialin staining. D) Sialin protein stain.*

**
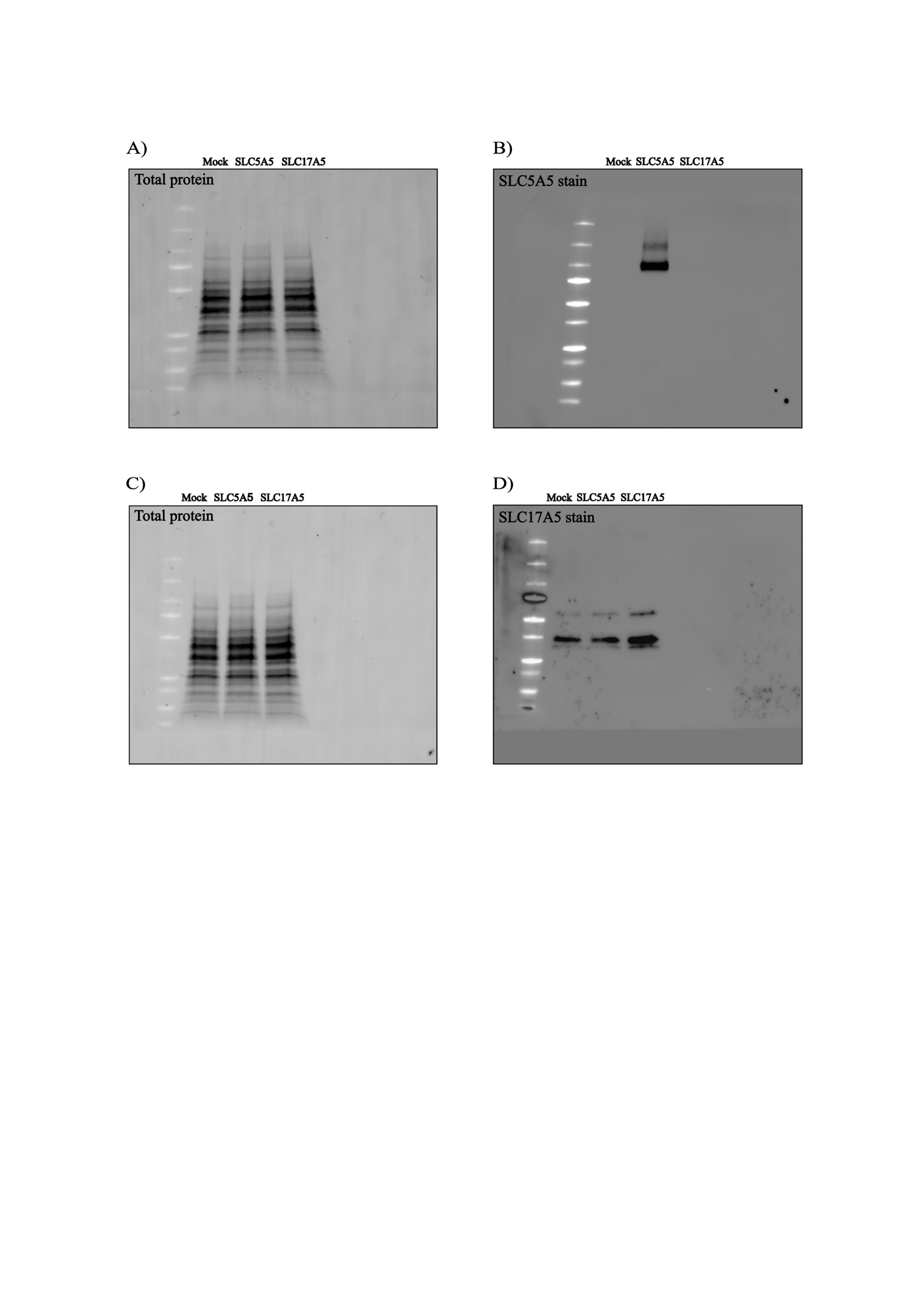
**

**Supplementary Table S4.** *Expression levels of slc5a5 and slc17a5 in stage V-VI X. laevis oocytes, comparing the two different X. Laevis subgenomes: L subgenome, retaining more ancestral genes, and S subgenome, undergoing greater gene loss and rearrangement.*

| Database | Gene | Species | Level of expression | Average | u.m. |
| --- | --- | --- | --- | --- | --- |
| Xenbase | *slc5a5* | X. Laevis L. | 3,27 | 1,63 | TPM |
| Xenbase | *slc5a5* | X. Laevis S. | Non detectable |  | TPM |
|  |  |  |  |  |  |
| Xenbase | *slc17a5* | X. Laevis S. | 6,33 | 4,6 | TPM |
| Xenbase | *slc17a5* | X. Laevis L. | 2,9 |  | TPM |

**Supplementary Figure 3. Nitrite does not evoke currents in SLC5A5 and SLC17A5-expressing oocytes.** *Application of 10 mM NaNO_2_ solution does not affect the transmembrane current in SLC5A5-injected oocytes or SLC17A5-injected oocytes, in blue. Holding membrane potential of -60 mV. Recordings were performed at -80 mV.*


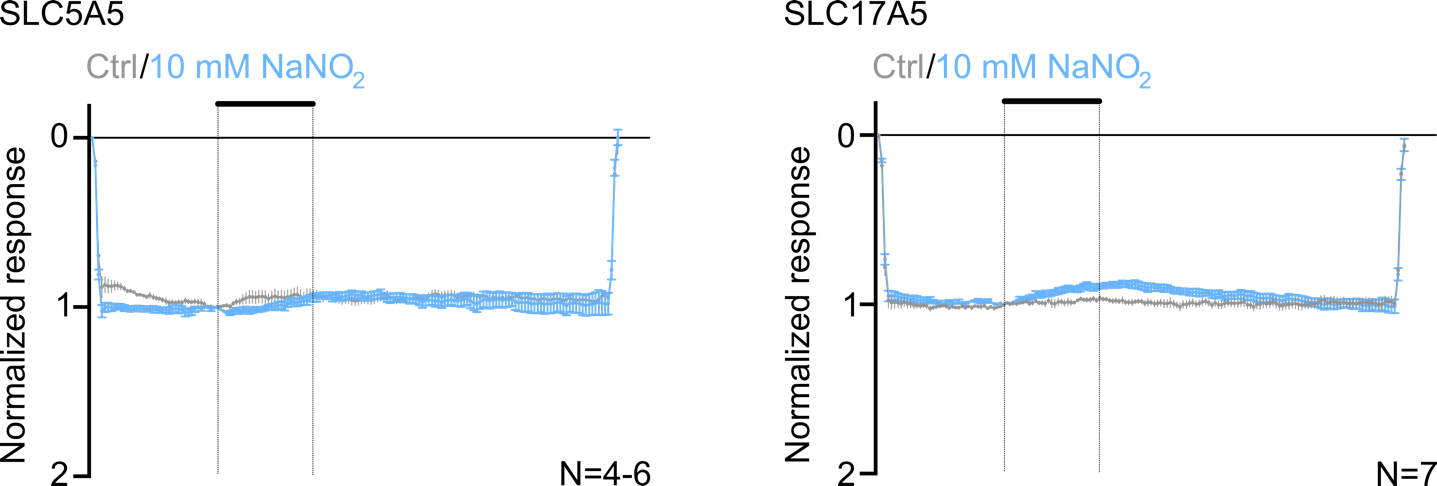


**Supplementary** **Figure 4.** *Brightfield and fluorescence microscopy confirmed comparable cell confluence across conditions (70–90%) and efficient transfection in and SLC5A5 and mock- transfected cells. Detectable eGFP fluorescence was observed in both SLC5A5-eGFP– and mock-transfected cells, while no fluorescence was detected in naïve cells, confirming the specificity of eGFP expression.*


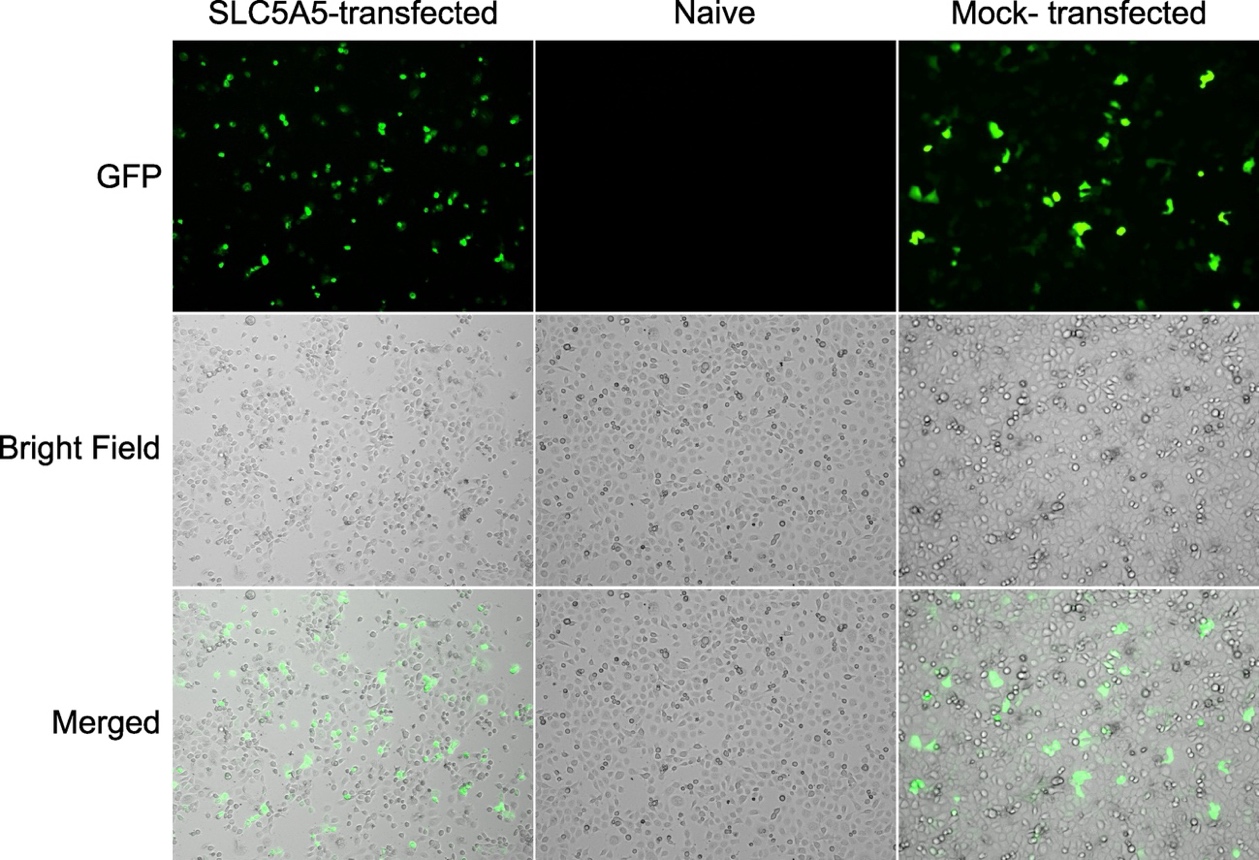


**Supplementary Figure 5.** *Uncropped total protein and protein stains from* ***Figure 6****. A) Stain-free image of total protein used for normalization of NIS staining. B) NIS protein stain. C) Stain-free image of total protein used for normalization of Sialin staining. D) Sialin protein stain.*

**
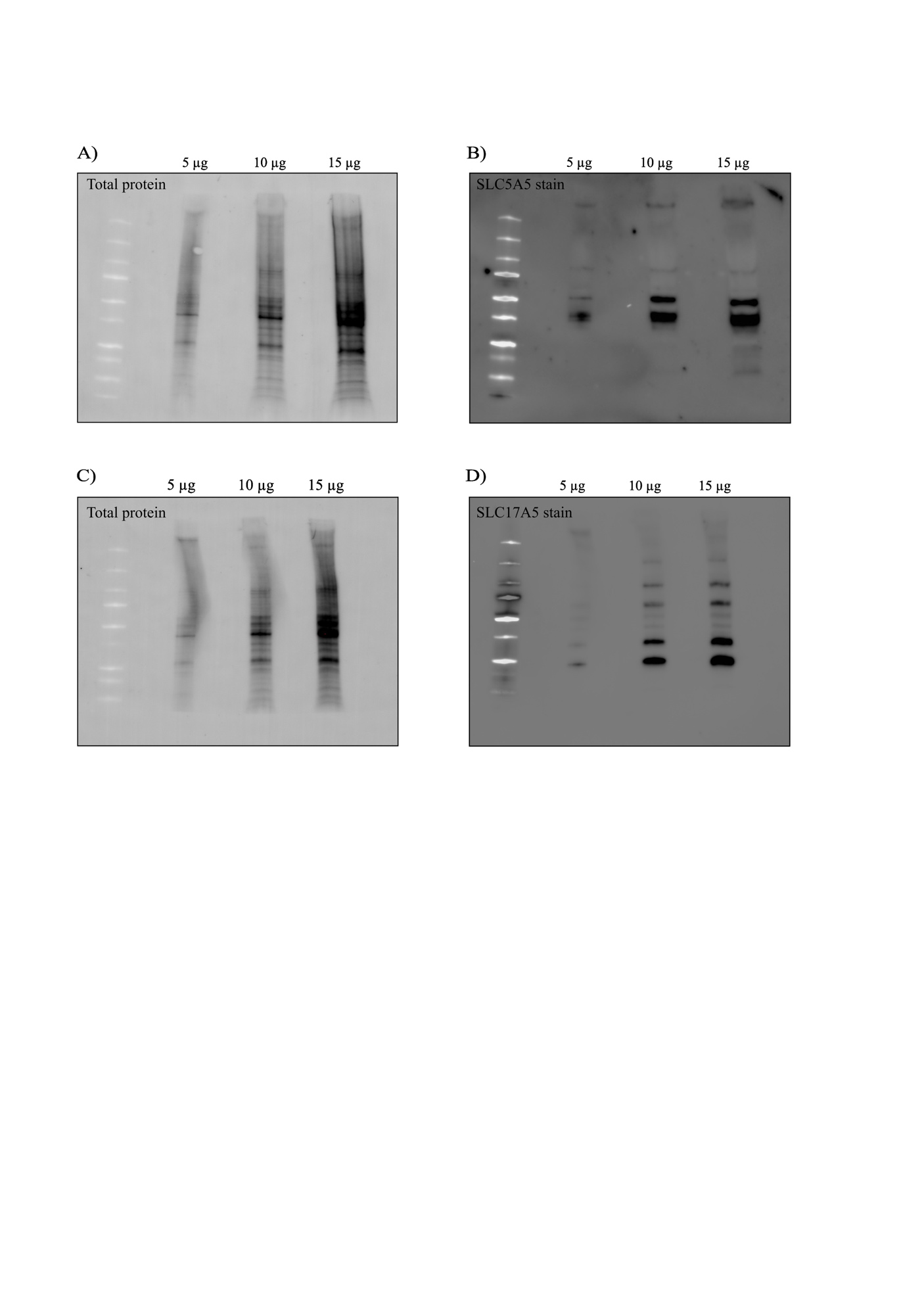
**

**Supplementary Table S5.** *Clinical overview of 15 patients, including demographic and clinical characteristics, contrast agent volume used during procedures, and renal function markers.*

| Age | Sex | Acute condition | Iomeron (ml) | Creatinine (µmol/L) | eGFR (ml/min) |
| --- | --- | --- | --- | --- | --- |
| 73 | male | NSTEMI | 255 | 107 | 53 |
| 71 | male | UA | 87 | 73 | 82 |
| 74 | female | UA | 550 | 67 | 68 |
| 80 | male | NSTEMI | 120 | 145 | 38 |
| 70 | male | NSTEMI, PCI after incomplete surgrey | 135 | 70 | 56 |
| 47 | male | Stable Angina | 108 | 71 | >90 |
| 63 | male | Stable Angina | 112 | 71 | >90 |
| 66 | male | Stable CAD | 289 | 73 | 67 |
| 68 | female | NSTEMI | 96 | 63 | 76 |
| 61 | male | Stable Angina | 142 | 84 | 75 |
| 68 | female | Stable Angina | 236 | 63 | 75 |
| 51 | male | Stable Angina | 160 | 76 | 82 |
| 56 | female | Stable Angina | 152 | 67 | 80 |
| 50 | female | Stable Angina | 85 | 59 | 90 |
| 85 | female | Aortic Stenosis | 146 | 74 | 56 |

NSTEMI: Non-ST-Elevation Myocardial Infarction, UA: Unstable Angina, PCI: Percutaneous Coronary Intervention, Stable CAD: Stable Coronary Artery Disease, eGFR: estimated Glomerular Filtration Rate

**Supplementary Table S6.** *Comparison between slc5a5 and slc17a5 in mus musculu from the Human Protein Atlas (proteinatlas.org).*

| Database | Gene | Species | Level of expression | u.m. |
| --- | --- | --- | --- | --- |
| Expression Atlas | *Slc5a5* | Mus musculus | 7 | TPM |
| Expression Atlas | *Slc17a5* | Mus musculus | 113 | TPM |

## TPM: Transcript Per Million
